# Differences in immune responses against *Leishmania* induced by infection and by immunization with killed parasite antigen: implications for vaccine discovery

**DOI:** 10.1186/s13071-016-1777-x

**Published:** 2016-09-06

**Authors:** Sergio C. F. Mendonça

**Affiliations:** Laboratório de Imunoparasitologia, Instituto Oswaldo Cruz, Fundação Oswaldo Cruz, Av. 4365 - Manguinhos, 21040-360 Rio de Janeiro, Brazil

**Keywords:** Immunity, Leishmaniasis, Antigens, Infection, Immunization, Vaccines

## Abstract

The leishmaniases are a group of diseases caused by different species of the protozoan genus *Leishmania* and transmitted by sand fly vectors. They are a major public health problem in almost all continents. There is no effective control of leishmaniasis and its geographical distribution is expanding in many countries. Great effort has been made by many scientists to develop a vaccine against leishmaniasis, but, so far, there is still no effective vaccine against the disease. The only way to generate protective immunity against leishmaniasis in humans is leishmanization, consisting of the inoculation of live virulent *Leishmania* as a means to acquire long-lasting immunity against subsequent infections. At present, all that we know about human immune responses to *Leishmania* induced by immunization with killed parasite antigens came from studies with first generation candidate vaccines (killed promastigote extracts). In the few occasions that the T cell-mediated immune responses to *Leishmania* induced by infection and immunization with killed parasite antigens were compared, important differences were found both in humans and in animals. This review discusses these differences and their relevance to the development of a vaccine against leishmaniasis, the major problems involved in this task, the recent prospects for the selection of candidate antigens and the use of attenuated *Leishmania* as live vaccines.

## Background

The leishmaniases are a group of vector-borne diseases which pose a major public health problem to many countries [1]. They are broadly classified as tegumentary (or cutaneous) and visceral leishmaniasis [2]. The former produces chronic lesions in the skin and, occasionally, in the naso-oral mucosa and in severe cases can lead to serious disfigurement [3]. The latter attacks lymphoid organs rich in mononuclear phagocytic cells, such as spleen, liver, bone marrow and lymph nodes and can lead to death if not treated [4]. It has been estimated that approximately 0.2−0.4 cases of visceral leishmaniasis and 0.7−1.2 million cases of tegumentary leishmaniasis occur each year globally, with 20,000−40,000 deaths per year due to visceral leishmaniasis, but these numbers are probably underestimated. Six countries (India, Bangladesh, Sudan, South Sudan, Ethiopia and Brazil) account for more than 90 % visceral leishmaniasis cases. Tegumentary leishmaniasis shows a wider geographical distribution, with the Americas, the Mediterranean basin, and western Asia being the most affected regions [1].

The infection is caused by different species of the protozoan genus *Leishmania* which are transmitted by a number of different sand fly (Phlebotominae) species to a variety of mammals, including man [5]. Diversity is thus the key word for defining the leishmaniases: a diversity of *Leishmania* species, sand fly vector species, eco-epidemiological conditions involved in transmission, and clinical presentations [6]. Besides that, there is a high degree of specificity in *Leishmania*-sand fly interactions [7] in the sense that each vector species typically transmits only one species of *Leishmania* [8]. It has been proposed that this species-specific vector competence is due to interspecies polymorphisms of lipophosphoglycan [9], a glycolipid highly abundant on the surface of *Leishmania* promastigotes [10], which mediates the attachment of their flagella to cells of the mid-gut epithelium of the vector [11]. The ecology and the habits of each specific vector create the particular conditions for transmission. That is why leishmaniasis can be either a zoonosis or an anthroponosis [12] and is transmitted to humans in sylvatic, domestic, and peridomestic cycles in ecosystems habitats ranging from cities to deserts and rain forests [5]. Moreover, currently used vector- and reservoir-targeted control strategies have not been successful [13, 14]. As a result of the absence of an effective control measure, the geographical distribution of leishmaniasis is continuously expanding to non-endemic areas, even reaching urban environments [15]. Due to all of these reasons, it is considered that a vaccine would be the most comprehensive and cost-effective tool for leishmaniasis control [16].

In spite of the global efforts on leishmaniasis vaccine development, there is still no effective vaccine against the human disease. At present, leishmanization is considered to be the only way to generate protective immunity against leishmaniasis in humans. This is an ancient practice from the Middle East. It consists on the inoculation of live virulent *Leishmania* in usually non-exposed areas of the body to avoid the development of lesions (and consequent scars) produced by natural infection in conspicuous sites. Its effectiveness is due to the immune protection conferred by the infection with *Leishmania major* against subsequent homologous infections. It is still used in a few countries, in spite of the obvious safety concerns [17, 18].

Although important differences between the immune responses induced by vaccines and infections have been found both in humans and in animals to various pathogens [19–27], there are very few studies addressing specifically this question with reference to leishmaniasis (Table 1). The aim of this review is to discuss the importance of these differences with regard to the development of a vaccine against leishmaniasis.

**Table 1:** Differences in immune responses induced by live parasites *vs* killed parasite antigens in leishmaniasis

Host species	*Leishmania* spp.	Experimental design	Major differences found	Reference
Mouse (C57BL/6 or BALB/c)	*L. major*	Early responses in draining lymph node cells were assessed after inoculation of 5 × 10^6^ live or killed *L. major* into naive mice	Cells from mice inoculated with killed parasites produced significantly more IL-4 and less IFN-γ than those from mice injected with live parasites	[24]
Mouse (C57BL/6)	*L. major*	Mice with healed *L. major* infections were compared to mice vaccinated with autoclaved *L. major* antigen + CpG ODN using infected sand fly challenge	Mice immunized with autoclaved *L. major* antigen + CpG ODN were protected against needle injection of parasites but not against infected sand fly challenge. On the other hand, mice that were healed from experimental *L. major* infections were highly resistant to sand fly transmitted infection	[42]
Human	*L. amazonensis*; *L, mexicana*; *L. guyanensis*; *L. major* (vaccine); *L. braziliensis* (infection)	PBMC from subjects immunized with a killed vaccine composed of five *Leishmania* strains and from ACL patients were stimulated in vitro with *L. braziliensis* crude antigen extract	The majority of the responding cells of the vaccinated subjects were CD8^+^ T cells, in contrast to the results of a group of patients, whose *Leishmania* antigen-reactive cells were mainly CD4^+^ T cells	[47]
Human	*L. amazonensis* (vaccine); *L. braziliensis* (infection)	The expression of Vβ chains of TCR was assessed in cells from ACL patients and healthy volunteers before and after vaccination	Vaccination led to a broad expansion of the TCR Vβ repertoire both in CD4^+^ and in CD8^+^ T cells. On the other hand, the patients showed a significant decrease in the expression of certain TCRs both in CD4^+^ and in CD8^+^ T cells when compared to healthy controls from the same leishmaniasis endemic area	[48]
Human	*L. amazonensis* (vaccine); *L. braziliensis* (infection)	Cytokine responses of PBMC from ACL patients and subjects immunized with a vaccine composed of killed promastigotes to La and to the recombinant protein LACK, both from *L. amazonensis*, were compared	The IFN-γ levels stimulated by La were significantly higher and the levels of IL-10 significantly lower than those stimulated by LACK in the patient group, whereas LACK induced a significantly higher IFN-γ production and a significantly lower IL-10 production compared with those induced by La in the vaccinated group LACK also induced a significantly higher frequency of IFN-γ-producing cells than did La in the vaccinated group	[49]

*Abbreviations*: *ACL* American cutaneous leishmaniasis, *CpG ODN* CpG oligodeoxynucleotides, *IFN-γ* Interferon- γ, *IL-10* interleukin-10, *La* whole-cell promastigote antigen extract, *LACK Leishmania* homologue of receptors for activated C kinase, *PBMC* peripheral blood mononuclear cells, *TCR* T-cell receptors

## Review

### Attempts to develop a vaccine against leishmaniasis

The leishmaniasis vaccine candidates can be broadly classified as first generation and second generation. First generation vaccine candidates are crude antigen extracts from killed promastigotes and have been used with or without BCG as adjuvant. An advantage of these vaccines is that they could be manufactured at low technological level and at relatively low cost in endemic countries [28]. However, their standardization would be an impossible task. In spite of the numerous clinical trials performed with these vaccines, their efficacy has not been clearly demonstrated [29]. Nevertheless, these trials have provided important information with regard to the human immune responses induced by immunization with *Leishmania* antigens [30]. On the other hand, the second generation candidates are based on chemically defined antigens and are generally produced using recombinant DNA technology. This group includes a variety of approaches for delivery of defined immunogens: recombinant proteins, DNA, and genetically engineered organisms, such as vectored vaccines and attenuated *Leishmania*. Since the content of the so-called second generation vaccines is precisely known, they tend to be more standardizable.

### Challenges for the development of a vaccine against leishmaniasis

There are important challenges to overcome for the development of a human leishmaniasis vaccine. The translation of the knowledge acquired from animal models to the real-life diseases and the transition from the basic research laboratory to the clinic has been largely unsuccessful [31]. The reductionist paradigm based on the *L. major* murine model may have played a role in this failure. Another important problem is that the adaptive immune response that confers protection against leishmaniasis is T cell-mediated [32]. According to Zinkernagel, all the available effective vaccines protect hosts via neutralizing antibodies (usually targeted to viruses or bacterial toxins), whereas for infections with intracellular pathogens, such as mycobacteria or *Leishmania*, which need T cell-mediated responses for their control, vaccines are either not available or not fully successful [33]. Another drawback is the absence of a reliable correlate of immune protection in leishmaniasis [31].

### Differences in anti-*Leishmania* immune responses induced by infection and immunization with killed parasite antigen

#### Studies in the mouse model

In the model of experimental infection of BALB/c mice with *L. major*, protective CD4^+^ T cell clones recognizing antigens associated with live parasites were generated [34], in contrast with previously described CD4^+^ T cell clones that recognized antigens from killed promastigotes and caused disease exacerbation [35]. More recently, the differences in the immune responses to live and killed *L. major* were addressed experimentally using BALB/c (susceptible) and C57BL/6 (resistant) mice. Although live and killed parasites were found to elicit comparable influx and/or proliferation of cells in the draining lymph nodes, the early cytokine responses to them were qualitatively different in both mouse strains. Cells from mice inoculated with live promastigotes produced significantly more interferon-gamma (IFN-γ) and less interleukin (IL)-4 in response to soluble *Leishmania* antigen in vitro than those from the mice inoculated with killed parasites. The use of CpG oligodeoxynucleotides (CpG ODN) as adjuvant changed the response of C57BL/6 mice injected with killed parasites to a predominantly IFN-γ response, which was shown to be protective against a subsequent virulent *L. major* challenge. However, the protection obtained with killed parasites plus CpG ODN was short-lived in comparison to that provided by the inoculation of live parasites [24].

In experimental murine leishmaniasis, it is well established that the same antigen can induce different kinds of immune responses, depending on the way that it is presented to the immune system. In the infection of BALB/c mice with *L. major*, the LACK antigen (*Leishmania* homologue of receptors for activated C kinase) induces an early production of IL-4 which initiates the development of a disease-promoting T helper type 2 (Th2) response [36], which causes the extreme susceptibility of these mice to this parasite [37]. In contrast, vaccination with LACK plus IL-12 [38], or with a DNA vaccine expressing LACK [39], is able to protect the same susceptible mice against *L. major* infection.

Another important issue concerning experimental infection is whether it is performed by needle injection or by sand fly bite. Several studies have shown remarkable differences between these two kinds of experimental challenge. It has been shown in *Leishmania mexicana*-infected *Lutzomyia longipalpis* that the transmission of parasites involves the regurgitation of a plug of promastigote secretory gel (PSG) that blocks the anterior parts of the midgut, where the metacyclic promastigotes accumulate. Filamentous proteophosphoglycan, a *Leishmania*-specific glycoprotein, was found to be the major component of PSG and to be responsible for disease exacerbation [40]. The same group has shown that immunization of BALB/c mice with *L. mexicana* PSG or with a synthetic vaccine containing the glycans found in it was able to protect them against challenge by the bites of infected sand flies but not against needle challenge [41]. Another interesting study has shown that C57BL/6 mice immunized with autoclaved *L. major* antigen + CpG ODN were protected against needle injection of parasites but not against infected sand fly challenge. However, mice that were healed from experimental *L. major* infections (transmitted by needle injection) were highly resistant to a subsequent sand fly-transmitted infection. It was shown by intra-vital microscopy and flow cytometry analysis that the infected sand fly bite, but not the needle challenge, resulted in a localized and sustained neutrophil recruitment at the inoculation site. Finally, it was possible to promote the efficacy of the killed vaccine by the removal of neutrophils after the sand fly challenge [42]. The suppressive effects of neutrophils on dendritic cells present in the dermis may be implicated in the incapacity of killed *Leishmania* vaccines to induce resistance against naturally transmitted infections [43]. Taken collectively, such findings provide evidence of the differences between infected sand fly and needle challenges [41, 42]. Nonetheless, it is noteworthy that the profile of the immune responses generated by vaccination with dead antigen is always very different from that resulting from the infection, regardless whether this is transmitted by sand fly bite or by needle injection.

#### Observations in humans

In the case of leishmaniasis, the relevance of studying the differences between the immune responses induced by live and killed parasites is obvious because leishmanization is the only effective way to achieve immune protection against leishmaniasis in humans [17] and so far this has not been possible with any human leishmaniasis vaccine [44].

A major challenge to the development of a leishmaniasis vaccine is the translation of the knowledge obtained in animal models to human disease [31]. Thus, understanding the human immune response towards the vaccine candidate molecules is indispensable for the development of a safe and effective vaccine. The human immune responses to defined *Leishmania* antigens have been studied almost exclusively in naturally infected (and sometimes naive) subjects [45]. With the single exception of the fusion recombinant protein LEISH-F1 (formerly known as Leish-111 F) [46], all that is known about human immune responses to *Leishmania* antigens induced by immunization came from studies with first generation candidate vaccines [30]. In the very few human studies in which immunization-induced immune responses were compared to those found in natural infection, profound differences between them have been revealed [47–49]. In subjects immunized with a first generation candidate vaccine made of killed promastigotes of five *Leishmania* strains, the majority of the cells responding to *Leishmania* antigens in vitro were found to be CD8^+^ T cells, in contrast to patients with active American tegumentary leishmaniasis, whose *Leishmania*-reactive T cells belonged mainly to the CD4^+^ phenotype [47]. A modulation on the TCR Vβ repertoire was found in American cutaneous leishmaniasis patients, who showed a significant lower expression of certain TCRs in both CD4^+^ and in CD8^+^ T cells as compared to age and gender matched healthy controls from the same leishmaniasis endemic area. On the other hand, immunization of human volunteers with a candidate vaccine containing killed *Leishmania amazonensis* promastigotes, led to a broad expansion of different Vβ TCRs. The authors concluded that their results indicate that infection with live parasites or exposure to antigens from killed promastigotes can differentially modulate the TCR Vβ repertoire [48]. Clear contrasts between the cytokine responses to *Leishmania* antigens primed by natural infection and by immunization with the same vaccine composed of killed *L. amazonensis* promastigotes have also been observed in peripheral blood mononuclear cells stimulated with crude whole-cell *L. amazonensis* promastigote extract (La) or with the recombinant protein LACK. In the group of patients with active cutaneous leishmaniasis the IFN-γ levels induced by stimulation with La were significantly higher and the levels of IL-10 significantly lower than those stimulated by LACK. However, LACK induced a significantly higher IFN-γ production and a significantly lower IL-10 production compared with those induced by La in the vaccinated group. LACK also induced a significantly higher frequency of IFN-γ-producing cells than did La in the vaccinated group [49]. The data described above indicate that immune responses to *Leishmania* antigens induced by live and killed parasites in men, as well as in mice [24, 34, 35, 42], are very different. In spite of the evidence of its importance for the identification of the factors involved in the generation of protective immunity and for the selection of potential vaccine candidates, this subject has been largely overlooked in leishmaniasis research.

### Selection of vaccine targets

In 1986, Mosmann et al. [50] identified two types of murine helper T cell clone characterized by different cytokine profiles and termed them Th1 and Th2, creating the so-called Th1/Th2 paradigm. These T helper cell subsets have found clear functional significance in the mouse model of *L. major* infection. While the resistant strains develop a healing Th1-type response after infection, the typically susceptible BALB/c mouse shows a Th2 response that leads to disease progression and eventually to death [51]. In this model, the key role played by IFN-γ and Th1 cells in the control of *Leishmania* infection has been clearly demonstrated [51, 52]. However, the clear-cut dichotomy found in the *L. major* model was not observed in the experimental infections of mice with other *Leishmania* species, namely those of the *Leishmania donovani* and *L. mexicana* complexes [53]. Nevertheless, this reductionist scheme has guided the understanding of immunopathology of the leishmaniases and the selection of candidate antigens for a leishmaniasis vaccine for several decades [54].

In an interesting review, Campos-Neto [54] reminded that antigens that stimulate a T helper type 1 (Th1) response during the disease or even after cure have shown no protective effect as a vaccine, while antigens associated with an early Th2 response have been found to be protective if a Th1 response to them is generated before infection. According to him, finding disease-associated Th2 antigens and inducing a Th1 immune response to them by vaccination could be a promising approach for developing a leishmaniasis vaccine [54]. Thus, considering that the immune responses to the same antigen induced by infection and immunization are different and sometimes even contrasting [49], trying to find antigens that induce a type 1 response in naturally infected subjects should not lead to the identification of a candidate antigen for an effective vaccine.

During coevolution, parasites have learned to inhibit or to subvert the host immune responses to their own benefit. Some *Leishmania* species are very effective in this regard [55, 56]. Particular parasite molecules play key roles in this successful adaptation. The expression of these molecules usually correlates with parasite infectivity and survival. That is why they are called virulence factors. At present, virulence factors are considered as potential drug targets and vaccine candidates for the control of leishmaniasis [57] and other infectious diseases [58]. A number of them have been identified in *Leishmania* spp. [2].

A possible way to identify immunogens with the potential to protect humans against leishmaniasis would be to compare immune responses of patients with active disease with those of vaccinated subjects. Using this strategy, we have found that the LACK antigen induced a proinflammatory cytokine response in peripheral blood mononuclear cells from vaccinated subjects, contrasting with that of cells from patients with active cutaneous leishmaniasis, which had significantly less IFN-γ and significantly more IL-10 than the former [49]. Antigens that are capable to induce regulatory (potentially disease-promoting) responses [59] in naturally infected patients and proinflammatory (potentially parasiticidal) [51, 52] responses in vaccinated subjects may be suitable for further investigations as candidates for a human vaccine. Immunological studies on human subjects immunized with first generation candidate vaccines can be useful in this regard. It should be reminded that some first generation candidate vaccines, such as Leishvacin®, have been shown to be safe [60] and immunogenic [61], although their efficacy has not been unambiguously established [62].

One could say that the *Leishmania* virulence factors already known have been identified almost accidentally, but now, following the systems immunology approach using the high-throughput methods currently available and with the help of advanced computational methods and bioinformatics infrastructures [63], the discovery of new virulence factors can be performed at a larger, systemic scale. Virulent and avirulent samples of the same parasite species or strain can be compared in order to identify differences in expression of multiple genes. In this sense, a very simple and efficient way to generate avirulent *Leishmania* is to keep promastigotes for long periods in axenic cultures [64].

Another interesting application for the identification of virulence factors would be the development of therapeutic vaccines and immunotherapy strategies. In this sense, Seifert et al*.* [65] have shown that a DNA vaccine candidate was able to increase the efficacy of a single suboptimal dose of liposomal amphotericin B in *L. donovani*-infected C57BL/6 mice. This DNA vaccine was composed of a mixture of five MIDGE-Th1 vectors encoding different leishmanial antigens. Interestingly, at least three of these five antigens have been characterized as virulence factors in *Leishmania*: kinetoplastid membrane protein-11 and the cysteine proteinases CPA and CPB [2, 6, 64, 66, 67]. Another therapeutic approach based on the neutralization of the effect of virulence factors has been proposed with the use of proteinase inhibitors for treating leishmaniasis [68].

### Live vaccines and concomitant immunity

Taking advantage of the better efficacy of live parasites to induce long-term protective immune responses as compared to killed parasite vaccines, the generation of attenuated parasites through targeted disruption of virulence factor coding genes is a contemporary approach for leishmaniasis vaccine development that is currently under intensive study [18]. In spite of the many problems that need to be circumvented before the efficacy of live attenuated vaccines can be assessed in clinical trials, namely safety, genetic stability, lack of transmissibility, preservation conditions, and limited persistence [69], this seems to be a promising prospect. Persistence of parasites is a key issue concerning the employment of attenuated *Leishmania* as vaccines. It has been shown that the sterile cure seen in IL-10-deficient mice [70] is followed by the loss of immunity to reinfection [71]. Therefore, parasite persistence should be involved in the long-lasting immunity provided by leishmanization [17, 72]. As far as live *Leishmania* vaccines are concerned, this issue needs to be more intensively investigated [73].

To understand why infection protects against reinfection and immunization with killed vaccines does not, the key factors for the development of concomitant immunity should be identified. Some light has recently been shed on this question. New data indicate that the protection conferred by concomitant immunity relies on the early presence (within 24 h) of IFN-γ producing CD4^+^ T cells, which are either rapidly recruited [74] or resident in the skin [75]. Peters et al. [74] used C57BL/6 mice clinically healed but chronically infected with *L. major* to demonstrate that concomitant immunity is mediated by short-lived CD44^+^CD62L^-^T-bet^+^Ly6C^+^CD4^+^ effector T cells that pre-exist secondary challenge and not by memory cells. According to the authors, these effector T cells are maintained at high frequencies during chronic infection via reactivation of central memory CD4^+^ T cells and the effector T cells themselves. Thus, the role of Th1 central memory T cells during chronic infection may not be to generate effector T cells following secondary challenge, but rather, to generate these Ly6C^+^ effector T cells prior to secondary challenge leading to effective concomitant immunity. This could be the reason for the failure of non-living vaccines to protect against *Leishmania* infections transmitted by sand flies. In another study, also performed with C57BL/6 mice clinically healed from *L.* major infection, skin-resident CD4^+^ T cells were found in the skin far from the site of the primary infection and were able to enhance protection against a later challenge by producing IFN-γ and recruiting circulating T cells to the skin in a CXCR3-dependent manner [75].

Taken together, all these data indicate that the best way to achieve protective immunity against *Leishmania* infection by vaccination should be with live vaccines, such as attenuated parasites. However, in this case, the long-time persistence of the parasites in the vaccinated subjects brings the concern of reversion to the pathogenic phenotype. An alternative would be to use a vaccination strategy able to generate long-lived memory CD4^+^ T cells and to keep the antigenic stimulation by repeated boosting or long-term antigen depots [74].

Finally, bearing in mind the diversity of the leishmaniases as a group of different diseases, it should be also considered that although resistance to reinfection has been clearly demonstrated both in the mouse model and in human leishmaniasis caused by *L. major*, this might not be the case in American tegumentary leishmaniasis [76].

## Conclusions

Currently, there is no effective measure to control any form of human leishmaniasis. For this reason, the geographical distribution of these diseases is expanding in many countries to new areas and even to cities. The absence of any effective control tool and the extreme diversity of the epidemiological factors involved in the transmission have led to the general opinion that a safe and effective vaccine would be the most comprehensive and cost-effective way to achieve leishmaniasis control.

Despite decades of effort by many research groups to develop a vaccine against leishmaniasis, no effective vaccine is yet available against human leishmaniasis. The only recognized way to generate protective immunity against leishmaniasis in humans is leishmanization, meaning the deliberate infection with live virulent parasites. These facts point to the importance of understanding the differences in the anti-*Leishmania* immune responses induced by infection and immunization for the development of an effective vaccine against leishmaniasis. In spite of the obvious relevance of this subject, there are surprisingly few studies addressing this question. All of them have revealed significant differences in the immune responses to *Leishmania* antigens primed by live parasites and killed parasite antigens in men and in mice.

These differences should be considered for the selection of vaccine candidate antigens. During many million years of coevolution, parasites have learned how to deliver and present antigens to the host immune system in such a way that potentially protective responses are either inhibited or subverted. That is why trying to find antigens that induce a protective type 1 response during infection will not help the discovery of promising vaccine candidates. Instead, the best vaccine candidates should be the antigens that promote disease by inducing pathogenic mechanisms during infection, or virulence factors, provided that a protective immune response could be induced against them by immunization protocols before infection. The concept of systems biology applied to immunology and the high-throughput techniques, which have been intensively developed over the last two decades, have made possible the identification of virulence factors in live parasites in large scale.

The evidence that live parasites are more able to induce long-term protective immune responses than killed antigen vaccines recommends the development of genetically engineered attenuated *Leishmania* to be used as live vaccines. Although this approach seems promising, there are many safety issues to be addressed before it can be tested in humans. The need for the presence of live parasites in order to maintain immunity will be a key issue in this regard, and deserves further investigation.

## Abbreviations

BCG: Bacillus Calmette-Guérin
CD: Cluster of differentiation
CPA: Cysteine proteinase A
CPB: Cysteine proteinase B
CpG ODN: CpG oligodeoxynucleotides
CXCR3: CXC chemokine receptor 3
DNA: Deoxyribonucleic acid
HIV: Human immunodeficiency virus
IFN-γ: Interferon-gamma
IL: Interleukin
La: Whole-cell *Leishmania amazonensis* promastigote extract
LACK: *Leishmania* homologue of receptors for activated C kinase
Leish-111 F (or LEISH-F1): *Leishmania*-derived recombinant polyprotein with three component proteins: thiol-specific antioxidant, *Leishmania major* stress-inducible protein 1 and *Leishmania* elongation initiation factor
Ly: Lymphocyte antigen
MIDGE: Minimalistic immunogenically defined gene expression
MPL-SE: Monophosphoryl lipid A plus squalene
PSG: Promastigote secretory gel
T-bet: T box expressed in T-cells
TCR Vβ: T cell receptor V beta
Th: T helper cell

## References

[1] Alvar J, Vélez ID, Bern C, Herrero M, Desjeux P, Cano J (2012). Leishmaniasis worldwide and global estimates of its incidence. PLoS One.

[2] Silva-Almeida M, Pereira BA, Ribeiro-Guimarães ML, Alves CR (2012). Proteinases as virulence factors in *Leishmania* spp. infection in mammals. Parasit Vectors.

[3] Goto H, Lindoso JA (2010). Current diagnosis and treatment of cutaneous and mucocutaneous leishmaniasis. Exp Rev Anti Infec Ther.

[4] Faleiro RJ, Kumar R, Hafner LM, Engwerda CR (2014). Immune regulation during chronic visceral leishmaniasis. PLoS Negl Trop Dis.

[5] Bern C, Maguire JH, Alvar J (2008). Complexities of assessing the disease burden attributable to leishmaniasis. PLoS Negl Trop Dis.

[6] Mendonça SCF, Cysne-Finkelstein L, Matos DCS (2015). Kinetoplastid membrane protein-11 as a vaccine candidate and a virulence factor in *Leishmania*. Front Immunol.

[7] Bates PA, Depaquit J, Galati EA, Kamhawi S, Maroli M, McDowell MA (2015). Recent advances in phlebotomine sand fly research related to leishmaniasis control. Parasit Vectors.

[8] Bates PA (2007). Transmission of *Leishmania* metacyclic promastigotes by phlebotomine sand flies. Int J Parasitol.

[9] Sacks DL (2001). *Leishmania*-sand fly interactions controlling species-specific vector competence. Cell Microbiol.

[10] Turco SJ (1988). The lipophosphoglycan of *Leishmania*. Parasitol Today.

[11] Sacks DL, Saraiva EM, Rowton E, Turco SJ, Pimenta PF (1994). The role of the lipophosphoglycan of *Leishmania* in vector competence. Parasitology.

[12] Lysenko AJ (1971). Distribution of leishmaniasis in the Old World. Bull World Health Organ.

[13] Werneck GL, Costa CH, de Carvalho FA, Pires e Cruz MS, Maguire JH (2014). Effectiveness of insecticide spraying and culling of dogs on the incidence of *Leishmania infantum* infection in humans: a cluster randomized trial in Teresina, Brazil. PLoS Negl Trop Dis.

[14] Moreira ED, de Souza VM M, Sreenivasan M, Nascimento EG, Pontes de Carvalho L (2004). Assessment of an optimized dog-culling program in the dynamics of canine *Leishmania* transmission. Vet Parasitol.

[15] Martins-Melo FR, Lima Mda S, Ramos AN, Alencar CH, Heukelbach J (2014). Mortality and case fatality due to visceral leishmaniasis in Brazil: a nationwide analysis of epidemiology, trends and spatial patterns. PLoS One.

[16] Sharifi I, FeKri AR, Aflatonian MR, Khamesipour A, Nadim A, Mousavi MR (1998). Randomised vaccine trial of single dose of killed *Leishmania major* plus BCG against anthroponotic cutaneous leishmaniasis in Bam, Iran. Lancet.

[17] Khamesipour A, Dowlati Y, Asilian A, Hashemi-Fesharki R, Javadi A, Noazin S (2005). Leishmanization: use of an old method for evaluation of candidate vaccines against leishmaniasis. Vaccine.

[18] Saljoughian N, Taheri T, Rafati S (2014). Live vaccination tactics: possible approaches for controlling visceral leishmaniasis. Front Immunol.

[19] Zhu X, Stauss HJ, Ivanyi J, Vordermeier HM (1997). Specificity of CD8+ T cells from subunit-vaccinated and infected H-2b mice recognizing the 38 kDa antigen of *Mycobacterium tuberculosis*. Int Immunol.

[20] Wennerås C, Qadri F, Bardhan PK, Sack RB, Svennerholm AM (1999). Intestinal immune responses in patients infected with enterotoxigenic *Escherichia coli* and in vaccinees. Infect Immun.

[21] Vogel TU, Horton H, Fuller DH, Carter DK, Vielhuber K, O'Connor DH (2002). Differences between T cell epitopes recognized after immunization and after infection. J Immunol.

[22] Gaunson JE, Philip CJ, Whithear KG, Browning GF (2006). Age related differences in the immune response to vaccination and infection with *Mycoplasma gallisepticum*. Vaccine.

[23] Petritus PM, Burns JM (2008). Suppression of lethal *Plasmodium yoelii* malaria following protective immunization requires antibody-, IL-4-, and IFN-gamma-dependent responses induced by vaccination and/or challenge infection. J Immunol.

[24] Okwor I, Liu D, Uzonna J (2009). Qualitative differences in the early immune response to live and killed *Leishmania major*: Implications for vaccination strategies against Leishmaniasis. Vaccine.

[25] Trible BR, Ramirez A, Suddith A, Fuller A, Kerrigan M, Hesse R (2012). Antibody responses following vaccination versus infection in a porcine circovirus-type 2 (PCV2) disease model show distinct differences in virus neutralization and epitope recognition. Vaccine.

[26] Leung DT, Uddin T, Xu P, Aktar A, Johnson RA, Rahman MA (2013). Immune responses to the O-specific polysaccharide antigen in children who received a killed oral cholera vaccine compared to responses following natural cholera infection in Bangladesh. Clin Vaccine Immunol.

[27] Valentini D, Ferrara G, Advani R, Hallander HO, Maeurer MJ (2015). Serum reactome induced by *Bordetella pertussis* infection and Pertussis vaccines: qualitative differences in serum antibody recognition patterns revealed by peptide microarray analysis. BMC Immunol.

[28] Evans KJ, Kedzierski L (2012). Development of vaccines against visceral leishmaniasis. J Trop Med.

[29] Noazin S, Khamesipour A, Moulton LH, Tanner M, Nasseri K, Modabber F (2009). Efficacy of killed whole-parasite vaccines in the prevention of leishmaniasis: a meta-analysis. Vaccine.

[30] Noazin S, Modabber F, Khamesipour A, Smith PG, Moulton LH, Nasseri K (2008). First generation leishmaniasis vaccines: a review of field efficacy trials. Vaccine.

[31] Kedzierski L1, Zhu Y, Handman E (2006). *Leishmania* vaccines: progress and problems. Parasitology.

[32] Howard JG, Liew FY (1984). Mechanisms of acquired immunity in leishmaniasis. Philos Trans R Soc Lond B Biol Sci.

[33] Zinkernagel RM (2003). On natural and artificial vaccinations. Annu Rev Immunol.

[34] Müller I, Louis JA (1989). Immunity to experimental infection with *Leishmania major*: generation of protective L3T4+ T cell clones recognizing antigen(s) associated with live parasites. Eur J Immunol.

[35] Titus RG, Lima GC, Engers HD, Louis JA (1984). Exacerbation of murine cutaneous leishmaniasis by adoptive transfer of parasite-specific helper T cell populations capable of mediating *Leishmania major*-specific delayed-type hypersensitivity. J Immunol.

[36] Launois P, Maillard I, Pingel S, Swihart KG, Xénarios I, Acha-Orbea H (1997). IL-4 rapidly produced by V beta 4 V alpha 8 CD4+ T cells instructs Th2 development and susceptibility to *Leishmania major* in BALB/c mice. Immunity.

[37] Julia V, Rassoulzadegan M, Glaichenhaus N (1996). Resistance to *Leishmania major* induced by tolerance to a single antigen. Science.

[38] Mougneau E, Altare F, Wakil AE, Zheng S, Coppola T, Wang ZE (1995). Expression cloning of a protective *Leishmania* antigen. Science.

[39] Gurunathan S, Sacks DL, Brown DR, Reiner SL, Charest H, Glaichenhaus N (1997). Vaccination with DNA encoding the immunodominant LACK parasite antigen confers protective immunity to mice infected with *Leishmania major*. J Exp Med.

[40] Rogers ME, Ilg T, Nikolaev AV, Ferguson MA, Bates PA (2004). Transmission of cutaneous leishmaniasis by sand flies is enhanced by regurgitation of fPPG. Nature.

[41] Rogers ME, Sizova OV, Ferguson MA, Nikolaev AV, Bates PA (2006). Synthetic glycovaccine protects against the bite of *Leishmania*-infected sand flies. J Infect Dis.

[42] Peters NC, Kimblin N, Secundino N, Kamhawi S, Lawyer P, Sacks DL (2009). Vector transmission of *Leishmania* abrogates vaccine-induced protective immunity. PLoS Pathog.

[43] Ribeiro-Gomes FL, Sacks D (2012). The influence of early neutrophil*-Leishmania* interactions on the host immune response to infection. Front Cell Infect Microbiol.

[44] Modabber F (2010). Leishmaniasis vaccines: past, present and future. Int J Antimicrob Agents.

[45] Mendonça SC, Russell DG, Coutinho SG (1991). Analysis of the human T cell responsiveness to purified antigens of *Leishmania*: lipophosphoglycan (LPG) and glycoprotein 63 (gp 63). Clin Exp Immunol.

[46] Duthie MS, Raman VS, Piazza FM, Reed SG (2012). The development and clinical evaluation of second-generation leishmaniasis vaccines. Vaccine.

[47] Mendonça SC, De Luca PM, Mayrink W, Restom TG, Conceicao-Silva F, Da-Cruz AM (1995). Characterization of human T lymphocyte-mediated immune responses induced by a vaccine against American tegumentary leishmaniasis. Am J Trop Med Hyg.

[48] Clarêncio J, de Oliveira CI, Bomfim G, Pompeu MM, Teixeira MJ, Barbosa TC (2006). Characterization of the T-cell receptor Vbeta repertoire in the human immune response against *Leishmania* parasites. Infect Immun.

[49] Azeredo-Coutinho RB, Matos DC, Armôa GG, Maia RM, Schubach A, Mayrink W (2008). Contrasting human cytokine responses to promastigote whole-cell extract and the *Leishmania* analogue receptor for activated C kinase antigen of *L. amazonensis* in natural infection versus immunization. Clin Exp Immunol.

[50] Mosmann TR, Cherwinski H, Bond MW, Giedlin MA, Coffman RL (1986). Two types of murine helper T cell clone. I. Definition according to profiles of lymphokine activities and secreted proteins. J Immunol.

[51] Locksley RM, Heinzel FP, Sadick MD, Holaday BJ, Gardner KD (1987). Murine cutaneous leishmaniasis: susceptibility correlates with differential expansion of helper T-cell subsets. Ann Inst Pasteur Immunol.

[52] Moll H, Röllinghoff M (1990). Resistance to murine cutaneous leishmaniasis is mediated by TH1 cells, but disease-promoting CD4+ cells are different from TH2 cells. Eur J Immunol.

[53] McMahon-Pratt D, Alexander J (2004). Does the *Leishmania major* paradigm of pathogenesis and protection hold for new world cutaneous leishmaniases or the visceral disease?. Immunol Rev.

[54] Campos-Neto A (2005). What about Th1/Th2 in cutaneous leishmaniasis vaccine discovery?. Braz J Med Biol Res.

[55] Flora R, Aghazadeh-Dibavar S, Bandyopadhyay M, Dasgupta S (2014). Immunosuppression during *Leishmania donovani* infection: a potential target for the development of therapy. Ann Parasitol.

[56] Soong L (2012). Subversion and utilization of host innate defense by *Leishmania amazonensis*. Front Immunol.

[57] Mottram JC, Coombs GH, Alexander J (2004). Cysteine peptidases as virulence factors of *Leishmania*. Curr Opin Microbiol.

[58] Li S, Huang H, Rao X, Chen W, Wang Z, Hu X (2014). Phenol-soluble modulins: novel virulence-associated peptides of staphylococci. Future Microbiol.

[59] Sacks D, Anderson C (2004). Re-examination of the immunosuppressive mechanisms mediating non-cure of *Leishmani*a infection in mice. Immunol Rev.

[60] Marzochi KB, Marzochi MA, Silva AF, Grativol N, Duarte R, Confort EM (1998). Phase 1 study of an inactivated vaccine against American tegumentary leishmaniasis in normal volunteers in Brazil. Mem Inst Oswaldo Cruz.

[61] De Luca PM, Mayrink W, Pinto JA, Coutinho SG, Santiago MA, Toledo VP (2001). A randomized double-blind placebo-controlled trial to evaluate the immunogenicity of a candidate vaccine against American tegumentary leishmaniasis. Acta Trop.

[62] Vélez ID, Gilchrist K, Arbelaez MP, Rojas CA, Puerta JA, Antunes CM (2005). Failure of a killed *Leishmania amazonensis* vaccine against American cutaneous leishmaniasis in Colombia. Trans R Soc Trop Med Hyg.

[63] Furman D, Davis MM (2015). New approaches to understanding the immune response to vaccination and infection. Vaccine.

[64] Mukhopadhyay S, Sen P, Majumder HK, Roy S (1998). Reduced expression of lipophosphoglycan (LPG) and kinetoplastid membrane protein (KMP)-11 in *Leishmania donovani* promastigotes in axenic culture. J Parasitol.

[65] Seifert K, Juhls C, Salguero FJ, Croft SL (2015). Sequential chemoimmunotherapy of experimental visceral leishmaniasis using a single low dose of liposomal amphotericin B and a novel DNA vaccine candidate. Antimicrob Agents Chemother.

[66] Lacerda DI, Cysne-Finkelstein L, Nunes MP, De-Luca PM, Genestra MS, Leon LL (2012). Kinetoplastid membrane protein-11 exacerbates infection with *Leishmania amazonensis* in murine macrophages. Mem Inst Oswaldo Cruz.

[67] Alexander J, Coombs GH, Mottram JC (1998). *Leishmania mexicana* cysteine proteinase-deficient mutants have attenuated virulence for mice and potentiate a Th1 response. J Immunol.

[68] Pereira BA (2014). Souza-Silva F, Silva-Almeida M, Santos-de-Souza R, Gonçalves de Oliveira LF, Ribeiro-Guimarães ML, et al. Proteinase inhibitors: a promising drug class for treating leishmaniasis. Curr Drug Targets.

[69] Gannavaram S, Dey R, Avishek K, Selvapandiyan A, Salotra P, Nakhasi HL (2014). Biomarkers of safety and immune protection for genetically modified live attenuated leishmania vaccines against visceral leishmaniasis - discovery and implications. Front Immunol.

[70] Belkaid Y1, Hoffmann KF, Mendez S, Kamhawi S, Udey MC, Wynn TA (2001). The role of interleukin (IL)-10 in the persistence of *Leishmania major* in the skin after healing and the therapeutic potential of anti-IL-10 receptor antibody for sterile cure. J Exp Med.

[71] Belkaid Y, Piccirillo CA, Mendez S, Shevach EM, Sacks DL (2002). CD4^+^CD25^+^ regulatory T cells control *Leishmania major* persistence and immunity. Nature.

[72] Huang L, Hinchman M, Mendez S (2015). Coinjection with TLR2 agonist Pam3CSK4 reduces the pathology of leishmanization in mice. PLoS Negl Trop Dis.

[73] Dey R, Dagur PK, Selvapandiyan A, McCoy JP, Salotra P, Duncan R (2013). Live attenuated *Leishmania donovani* p27 gene knockout parasites are nonpathogenic and elicit long-term protective immunity in BALB/c mice. J Immunol.

[74] Peters NC, Pagán AJ, Lawyer PG, Hand TW, Henrique Roma E, Stamper LW (2014). Chronic parasitic infection maintains high frequencies of short-lived Ly6C + CD4+ effector T cells that are required for protection against re-infection. PLoS Pathog.

[75] Glennie ND, Yeramilli VA, Beiting DP, Volk SW, Weaver CT, Scott P (2015). Skin-resident memory CD4+ T cells enhance protection against *Leishmania major* infection. J Exp Med.

[76] Saravia NG, Weigle K, Segura I, Giannini SH, Pacheco R, Labrada LA (1990). Recurrent lesions in human *Leishmania braziliensis* infection-reactivation or reinfection?. Lancet.

